# Deciphering the Code: Stem Cell-Immune Function and Cardiac Regeneration

**DOI:** 10.3390/cells10030592

**Published:** 2021-03-08

**Authors:** Gustav Steinhoff

**Affiliations:** Reference and Translation Center for Cardiac Stem Cell Therapy, Department of Cardiac Surgery, University Medicine Rostock, Schillingallee 35, 18057 Rostock, Germany; gustavsteinhoff@med.uni-rostock.de; Tel.: +49-179-39-39-344

The development of stem-cell-based and regenerative therapies for cardiovascular and other diseases has faced an unexpected roadblock in clinical translation [1]. Emerging knowledge from clinical research points in the direction of bone marrow stem cell/immune progenitor cell senescence by epigenetic and somatic mutations being the potential cause [2]. Moreover, not only is the modification of tissue repair mechanisms the consequence of stem cell mutations and DNA-pathology, but these may also form the cause of disease mechanisms and progression [1,3].

To identify responsible pathways, genotype/phenotype patterns and establish proof by modeling, a close interdisciplinary approach is needed crossing medical and nonmedical disciplines. This concept is highly visible in the excellent contributions to this special issue. 

To unravel this challenging puzzle of disease diagnostics in stem/immune cells, there is a need for better-individualized diagnostics (precision medicine) of underlying disease mechanisms using the new technologies of systems medicine and nuclear medicine tissue imaging. In the Special Issue, stem cell senescence diagnostics in cardiovascular disease, diabetic heart disease, and amyotrophic lateral sclerosis are presented as well as the impact of immune reaction for cardiac regeneration. Validated precision T-cell targeted and (stem) cell therapies modified by mRNA can also be designed based on diagnostic monitoring of molecular pathomechanisms altered by genetic or cellular repair strategies. 

Several groups are helping to clear the roadblock using new technologies and precision medicine in clinical and experimental studies. This Special Issue places particular emphasis on stem cell/immune dysfunction in hematologic, cardiac, and neuronal disease based on immune and cardiovascular pathomechanisms. The first paper by Galow et al. enlightens the heavily debated question of cardiomyocyte renewal using single nuclei sequencing technology in inbred and outbred mouse strain models [4]. The identified proliferative cardiomyocyte subpopulation clearly supports the regeneration of cardiomyocytes by cytogenesis rather than progenitor cells. The second paper by Faulkner et al. unravels the cardioprotection reprogramming effect exerted by therapeutic application of the longevity-associated gene (LAV-BPIFB4) in diabetic mice cardiomyopathy using a multi-omics analysis [5]. Their finding of a boost of mitochondrial metabolic gene expression by LAV-BPIFB4 identifies a new cardioprotection pathway. The third paper by Lang et al. used a new PET-CT imaging approach with a [^68^Ga]-NODAGA-RGD tracer binding to alpha V-ß3 angiogenesis receptors in a mouse infarction model using cell transplantation of ES-derived cardiac-induced cells for the induction of cardiac regeneration [6]. Interestingly, tracer binding was reduced in mice receiving cell transplantation leading to enhanced regeneration. Another mouse model of cardiomyocyte transplantation from pluripotent embryonic stem cells was used for the treatment of chronic Chagas-cardiomyopathy by Brasil et al. [7]. Cardiac recovery, however, was not induced by cardiomyocyte cell transplantation in chronic Chagas cardiomyopathy. The therapeutic effect of intrathecal autologous Lin^-^ bone marrow stem cell transplantation was studied by Baumert et al. in a Phase I amyotrophic lateral sclerosis (ALS) trial [8]. Safety and efficacy were demonstrated, and multi-omics signatures for response and non-response were identified. Altered immune response was observed in post-myocardial infarction regeneration after cardiomyocyte transplantation in a mouse model by Vasudevan et al. [9]. Gene expression signatures for cardiac regeneration involved circadian regulation, mitochondrial metabolism, and immune responses after cardiomyocyte transplantation. The functional consequences of immune cell senescence and aging of the immune system have important consequences for heart function, as reviewed by Tobin et al. [10]. On this basis, rejuvenation of the aged immune system may be a valid therapeutic candidate to prevent or treat heart disease. For immune reconstitution, the success of engraftment of hematopoietic stem cells can be improved by targeting *Mapk14* (*p38*) was demonstrated by Klatt et al. [11]. An experimental approach for cell senescence protection to UV-light-induced senescence was demonstrated by Bellu et al. in skin stem cells using pretreatment with *Myrtus communis* natural extract combined with a polycaprolactone nanofibrous scaffold (NanoPCL-M) [12]. Stem cell repair and cardiovascular regeneration control by mRNA were reviewed by Chanda et al. [13]. This combined diagnostic and therapeutic approach to repair stem cell senescence and immune dysfunction is the main approach for next-generation cardiovascular stem cell therapy.
